# Mechanisms, costs, and carry‐over effects of cannibal‐induced developmental plasticity in invasive cane toads

**DOI:** 10.1002/ece3.10961

**Published:** 2024-02-09

**Authors:** Michael R. Crossland, Richard Shine, Jayna L. DeVore

**Affiliations:** ^1^ School of Life and Environmental Sciences A08 The University of Sydney Sydney New South Wales Australia; ^2^ School of Natural Sciences Macquarie University Sydney New South Wales Australia

**Keywords:** anuran, inducible defence, phenotypic plasticity, predation, trade‐off

## Abstract

Inducible defences can improve survival in variable environments by allowing individuals to produce defences if they detect predators. These defences are often expressed as inter‐related developmental, morphological, and behavioural changes. However, producing defences can incur costs, which may be expressed immediately and/or during subsequent life stages. In Australia, waterborne cues of potentially cannibalistic conspecific tadpoles induce hatchlings of invasive cane toads to accelerate their developmental rate, thereby reducing their window of vulnerability. However, the mechanisms and costs of such accelerated development are poorly understood, and whether cane toad embryos show cannibal‐induced plasticity in other traits is unknown. Here, we found no evidence of altered time of hatching for embryos exposed to non‐feeding conspecific cannibal tadpole cues. Additionally, hatchling dispersal behaviours were not affected by exposure to these cues. However, developmental acceleration of hatchlings induced by exposure to tadpole cues was accompanied by reduced hatchling growth, indicating a trade‐off between these processes. At the conclusion of the hatchling stage, cannibal‐exposed individuals were smaller and morphologically distinct from control siblings. This size reduction affected performance during the subsequent tadpole stage: smaller cannibal‐exposed individuals were more likely to die, and initial size tended to be positively associated with subsequent tadpole growth and development across treatments (respectively, *p* = .07 and *p* = .06). However, even accounting for variation in initial size, there was an additional negative effect of cannibal exposure on tadpole growth and development, demonstrating that the fitness costs associated with developmental acceleration are not entirely attributable to size reductions.

## Graphical abstract

Hatchlings of invasive cane toads in Australia respond to risk of cannibalism by conspecific tadpoles by accelerating development. This occurs as a trade‐off with growth. The resulting size reduction predicts some, but not all, future fitness costs.

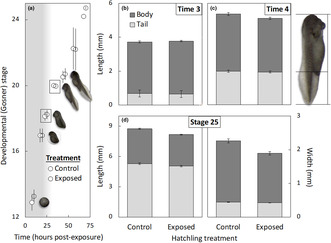

## 1 INTRODUCTION

Phenotypic plasticity, the ability of a single genotype to produce different phenotypes, physiological states, and/or behaviours in response to prevailing environmental conditions, is a common trait in many organisms (Beldade et al., 2011; Reger et al., 2018; Stearns, 1989; West‐Eberhard, 1989). This flexibility can allow organisms to better deal with heterogeneous environments (adaptive plasticity); however, plasticity is presumed to come with costs and limitations (Snell‐Rood et al., 2010; Stearns, 1989; Taborsky, 2017) and not all plastic responses are adaptive (Ghalambor et al., 2007; Stearns, 1989). Developmental plasticity is a form of phenotypic plasticity, whereby individuals adjust their developmental rate and/or trajectory in response to environmental conditions experienced during ontogeny (Beldade et al., 2011; Taborsky, 2017). Developmental plasticity is widespread among taxonomic groups (Benard, 2004; Taborsky, 2017; Warkentin, 2011a) and can occur in response to a variety of abiotic (nutrition, photoperiod, temperature, pH, salinity) and biotic (conspecific cues, predator cues) factors (Beldade et al., 2011; Benard, 2004; Taborsky, 2017) and for some traits can be irreversible (Kotrschal & Taborsky, 2010; Taborsky, 2006, 2017).

Predation has been particularly well documented as a powerful force driving the evolution and expression of developmental plasticity (Beldade et al., 2011; Reger et al., 2018; Vila Pouca et al., 2021), especially for organisms with complex life‐histories such as amphibians (Benard, 2004; Warkentin, 2011b). Adaptive predator‐induced plasticity generally optimises fitness by allowing individuals to invest in producing defences against predation only if predators are detected. Predator‐induced plastic responses can be expressed in a multitude of ways, including adaptive changes in behaviour, morphology, or the timing of life stage transitions; such responses occur rapidly in a wide variety of animal species (Benard, 2004; Ferrari et al., 2010). For example, in anurans, American toad tadpoles reduce activity (Relyea, 2001) and chorus frog tadpoles produce larger tails (Van Buskirk et al., 1997) in response to olfactory cues from predatory dragonfly larvae, and red‐eyed treefrogs hatch earlier in development in response to vibrational cues from ovivorous snakes (Warkentin, 1995). In some cases, these adaptive responses are complex, and both behavioural and morphological changes contribute to reducing predation risk. For example, wood frog larvae develop larger tails and smaller bodies, and decrease their activity, in response to cues from predatory dragonfly larvae (Relyea, 2004). However, inducible defences are generally produced at a cost (e.g., these predator‐induced morphological changes in wood frog tadpoles also reduce digestive efficiency and growth: Relyea & Auld, 2004).

Both behavioural and morphological responses have energetic trade‐offs, but behavioural responses may be more readily induced than morphological responses due to the higher costs and delayed responses associated with the latter, combined with often‐unpredictable temporal and spatial variation in predation risk (Ferrari et al., 2010). The costs associated with plastic responses can reduce the adaptive value of the response; this may be especially so if induced in an environment where predators are actually absent and there is no immediate predation risk, such as prey continuing to respond to historic encounters with predators or lingering predator cues when those predators are no longer present (Boissy, 1995; Fraker, 2009; Gabriel et al., 2005; Van Buskirk, 2002). However, in cases where predation risk is extreme, even costly responses that reduce predation risk can still be adaptive. In some cases, the costs of inducing a defensive response during one life history stage continue to affect predator‐exposed individuals during subsequent life history stages. For example, slower growth during the tadpole stage as a result of predator exposure can reduce size at metamorphosis, lowering the probability of survival in the terrestrial environment (Relyea, 2007). Exposure to predation risk during one life stage can therefore continue to affect an individual throughout its ontogeny. Another factor that can influence how strongly an individual is affected by exposure to predation risk is the ontogenetic timing of this exposure. Exposure to predation in early development may have especially strong effects (Frankenhuis & Panchanathan, 2011), as early alterations in developmental trajectories can continue to affect impacted individuals at later life stages. The severity of the threat and the timing of the exposure therefore both influence the degree to which an individual is likely to be affected by predation risk.

One system in which individuals often experience fluctuating degrees of predation risk during an early life stage is the targeted cannibalism of recently deposited cane toad clutches by conspecific tadpoles in Australia. For these clutches, cannibalism by older cohorts of tadpoles is the primary risk of mortality. This risk is immediate and extreme: cannibalistic toad tadpoles can not only consume toad eggs that they encounter by chance, they also rapidly locate recently hatched, immobile, pre‐feeding larvae (hatchlings) after their emergence from the egg capsule using chemical cues (maternally invested defensive toxins) exuded by the hatchlings (Crossland et al., 2021, 2022; Crossland & Shine, 2011). These toxin cues not only attract older conspecific tadpoles, they also trigger a change in behaviour of these tadpoles by inducing grazing (Crossland & Shine, 2011). As a result, tadpoles exposed to hatchling cues not only become highly cannibalistic but they also consume prey such as native anuran eggs which they normally disregard (Crossland et al., 2022). Despite the relatively short duration of the vulnerable egg and hatchling life stages (~3–5 days), cannibalism by older tadpoles during this time frequently results in clutch mortality rates of >99% in natural waterbodies (Alford et al., 1995; DeVore, Crossland, & Shine, 2021). Thus, selection pressure for hatchlings to minimise cannibalism is intense. In response to this risk, most cane toad clutches in Australia exhibit a facultative plastic response: when exposed to waterborne chemical cues of conspecific tadpoles, these clutches accelerate their development rate in the late hatchling stages to more quickly reach the free‐swimming tadpole stage that is not vulnerable to cannibalism (DeVore, Crossland, & Shine, 2021; DeVore, Crossland, Shine, & Ducatez, 2021). This developmental acceleration occurs even when the conspecific tadpoles used to induce the response have been raised on a diet of algal wafers and fasted during the exposure period (DeVore, Crossland, & Shine, 2021; DeVore, Crossland, Shine, & Ducatez, 2021). This contrasts with the predator‐induced responses of some anuran species for which responses depend on the diet of the predator. For example, tadpoles of the Gray Treefrog (*Hyla versicolor*) show strong behavioural responses when exposed to chemical cues of predatory dragonfly naiads (*Anax junius*) that have previously consumed conspecific and heterospecific amphibian prey but show no response to cues from starved naiads (Schoeppner & Relyea, 2009). This difference is likely because, for cane toad hatchlings in Australia, chemical cues from conspecific tadpoles inherently indicate a high risk of cannibalism; even tadpoles that have never encountered hatchlings before become highly cannibalistic upon detecting chemical cues from hatchlings (DeVore, Crossland, & Shine, 2021; DeVore, Crossland, Shine, & Ducatez, 2021). However, interestingly, there is significant variation among cane toad clutches in this inducible hatchling response. Clutches that inherently develop slowly exhibit developmental acceleration when exposed to conspecific tadpole cues, while clutches that inherently develop quickly do not accelerate development when exposed to these cues, possibly because they have reached their physiological limit (i.e., development rate has become canalised) and so cannot accelerate development further (DeVore, Crossland, & Shine, 2021).

Although the developmental rate of cane toad hatchlings in Australia has been demonstrated to be a plastic trait in response to conspecific tadpole cues, it is not known whether tadpole‐exposed clutches also exhibit adaptive inducible defences in other developmental traits. For example, a number of amphibian species can alter timing of egg hatching in response to predation risk by either hatching early (Capellán & Nicieza, 2007; Chivers et al., 2001; Delia et al., 2019; Gomez‐Mestre et al., 2008; Laurila et al., 2002; Poo & Bickford, 2014; Warkentin, 1995) or by delaying hatching (Gazzola et al., 2015; Moore et al., 1996; Schalk et al., 2002; Sih & Moore, 1993). In cane toads, adaptive changes in the timing of hatching could also reduce predation risk, especially since the cues that attract and induce cannibalistic behaviours in tadpoles are only detectable after the jelly layers disintegrate and the hatchlings emerge from the egg capsule (Crossland et al., 2022; Crossland & Shine, 2011). If toad embryos respond to cannibal cues by delaying emergence from the egg capsule (and hatching at a more advanced developmental stage), they could reduce the duration of the hatchling life stage that actively attracts cannibalistic tadpoles. Morphological plasticity could also contribute to reducing cannibalism risk: although cane toad hatchlings are relatively immobile prior to reaching the tadpole phase, they are capable of twitching and (later in development) bursts of swimming. The induction of morphological features that facilitate swimming, such as larger tails, could therefore also be favoured in response to cannibal cues. Behavioural changes in response to predation risk are also frequently an important component of inducible defences (Relyea, 2001). Inducible behavioural responses are thought to be especially effective because they can be rapidly induced when a threat is detected, minimising the time lag between the detection of the threat and the expression of the adaptive response. Behavioural responses that could minimise cannibalism risk in cane toads are the rate at which hatchlings (a) disperse from the egg string (thereby potentially dispersing away from the chemical cues used by cannibal tadpoles to target this life stage; such chemicals are likely to be at particularly high concentrations in the immediate vicinity of the egg string as large numbers of hatchlings emerge within a very short time period), and/or (b) transition to an upright body posture (enabling dispersal as well as faster escape responses when physically contacted by cannibalistic tadpoles).

Exposure to non‐feeding cannibal tadpole cues during the vulnerable egg and hatchling life stages generally also results in significant carry‐over effects of reduction in growth, development, and survival in subsequent cane toad tadpole stages (Clarke et al., 2015, 2016; Crossland & Shine, 2012; DeVore, Crossland, & Shine, 2021; DeVore, Crossland, Shine, & Ducatez, 2021; McCann et al., 2020). These carry‐over effects only occur following exposure during egg and hatchling stages; free‐swimming cane toad tadpoles (that are not vulnerable to cannibalism) exposed to older conspecific tadpole cues do not experience such carry‐over effects (Clarke et al., 2015). Thus, accelerated development of hatchlings exposed to older conspecific tadpole cues has been interpreted as a response to minimise cannibalism risk (DeVore, Crossland, & Shine, 2021). The negative carry‐over effects following embryonic exposure to conspecific tadpole cues, combined with the recent discovery that hatchlings can accelerate development rate when exposed to these cues, suggests that the adaptive plastic response of developmental acceleration is associated with strong future costs. However, the mechanisms that underlie these performance reductions are not understood. One possibility is that facultative developmental acceleration of cane toad hatchlings occurs as a trade‐off with hatchling growth, and that size reductions at the conclusion of the hatchling stage affect fitness during the subsequent tadpole life history stage. Growth/development trade‐offs are well known for the tadpole stage of anurans (Bridges, 2002; Kulkarni et al., 2011; Wilbur & Collins, 1973), although predator‐induced changes in tadpole feeding behaviour generally make it difficult to determine whether producing a given developmental defence is energetically costly (Benard, 2004; Relyea, 2007). In contrast, the potential for growth/development trade‐offs has not been explored during the hatchling stage of anurans, despite the pre‐feeding hatchling period being an ideal time to study these costs because eggs and hatchlings are entirely dependent on endogenous energy. This energy can be directed toward differentiation or growth; where energy resources are limited, there may be a trade‐off between these processes, such that accelerating development may only be achievable via reductions in growth. If this is the case, the resulting reductions in initial size at the beginning of the tadpole stage may contribute to performance reductions during the tadpole stage. Previous research has highlighted the possibility of such a trade‐off by demonstrating that hatchlings exposed to conspecific tadpoles often exhibit reductions in mean mass at the conclusion of the hatchling stage (DeVore, Crossland, & Shine, 2021), but it remains unclear whether energetic investment in increased dispersal behaviours in exposed treatments contributes to this effect, how morphological changes are involved, or whether resulting individual‐level variation in initial size affects performance during the subsequent life stage.

In this study, we present data from two experiments that assessed these possibilities, using cane toad clutches known to exhibit cannibal‐induced developmental plasticity (i.e., accelerate development rate in response to non‐feeding cannibal tadpole cues). The first experiment assessed four questions: (1) do toad embryos alter time of hatching in response to cannibal tadpole cues?, (2) does exposure to cannibal tadpole cues affect the morphology of developing hatchlings?, (3) does cannibal‐induced accelerated development of hatchlings occur at the expense of hatchling growth, indicating a growth/development trade‐off within the hatchling stage?, and (4) if so, are cannibal‐induced size reductions at the end of the hatchling stage linked to fitness reductions during the subsequent tadpole life history stage? The data to assess these four questions were collected as part of a larger study by DeVore, Crossland, and Shine (2021; see below for further details) but have not been published previously, as this previous study did not assess hatching time and success, morphological traits, or the relationship between individual‐level variation in size at the conclusion of the hatchling stage and subsequent performance during the tadpole stage. The second experiment was conducted separately and assessed whether hatchlings exposed to cannibal tadpole cues alter their behaviour by measuring (1) the rate of dispersal from the egg string and (2) the rate of transition from initial prone body posture upon hatching to upright body position.

## 2 MATERIALS AND METHODS

The cane toad (*Rhinella marina*, Linnaeus 1758) is a large (to >1 kg) anuran native to South America, that was introduced to Australia in 1935 as a biocontrol agent (Lever, 2001). It continues to spread across the Australian continent and poses a risk to native predator species due to its toxic chemical defences (Shine, 2010). A summary of the egg and larval life history stages is provided in Alford et al. (1995) and DeVore, Crossland, and Shine (2021). Briefly, cane toads breed in temporary and permanent water bodies, laying clutches of up to 40,000 eggs that are deposited in a long string. Hatching occurs at Gosner (1960) stage 17–18 (~48 h after egg deposition), with hatchlings developing into feeding, free‐swimming tadpoles (stage 25) another 24–48 h later. Tadpoles are omnivorous but are highly cannibalistic on younger cohorts of eggs and hatchlings (Alford et al., 1995; Crossland et al., 2022; Crossland & Shine, 2011; DeVore, Crossland, & Shine, 2021; DeVore, Crossland, Shine, & Ducatez, 2021). Development to metamorphosis is strongly influenced by density and temperature and can be rapid (~16 days) under optimal conditions.

### 2.1 Husbandry and experimental procedures

Adult cane toads were collected by hand from the wild in northern Australia and housed at the Tropical Ecology Research Facility, Northern Territory (12°34′43″ S, 131°18′51″ E) in outdoor bins (1 m × 1 m × 0.8 m) with refugia, water, and constant food supply. We induced toads to spawn by subcutaneous injection of synthetic gonadotrophin leuprorelin acetate (Lucrin, Abbot Australasia, 0.25 mg mL^−1^). Male toads were injected with 0.25 mL and female toads with 0.75 mL. Pairs of toads were placed in 80 L plastic tubs with a small amount of water and allowed to spawn overnight. The resultant eggs were transferred to 18 L plastic tubs filled with 9 L water, constantly aerated. Once embryos developed into free‐swimming tadpoles they were transferred to outdoor 750 L bins and fed algae wafers (Hikari, Kyorin, Japan) with weekly water changes. Eggs and tadpoles were haphazardly selected within clutches for experiments, as required.

The tadpoles used to generate cannibal tadpole cues in the experiments were Gosner (1960) stage 30–37 and were 47–55 days old (since egg deposition). We did not feed these tadpoles during experiments to be consistent with the methodology used in all previous studies examining effects of embryonic exposure to chemical cues of cane toad tadpoles (Clarke et al., 2015, 2016; Crossland & Shine, 2012; DeVore, Crossland, & Shine, 2021; DeVore, Crossland, Shine, & Ducatez, 2021; McCann et al., 2020), and to ensure that hatchling responses were induced by exposure to tadpole cues rather than by exposure to alarm cues from consumed hatchlings. Additionally, DeVore, Crossland, and Shine (2021) demonstrated that developmental acceleration of cane toad hatchlings occurs in response to chemical cues from fasted conspecific tadpoles (in contrast to other anuran species that do not respond to cues from starved predators; e.g., Schoeppner & Relyea, 2009). Despite the fact that the fasted tadpoles used to induce hatchling responses to chemical cues had not consumed conspecific hatchlings, we interpreted hatchling responses as “cannibal‐induced”, due to the fact that, in Australia, hatchling cues consistently induce cannibalistic behaviours in tadpoles (DeVore, Crossland, & Shine, 2021; see Section 4 for further comments). All water used in holding tanks and experimental containers was groundwater sourced from a local aquifer. All developmental stages reported below for eggs, hatchlings, and tadpoles are based on the staging system of Gosner (1960).

### 2.2 Experiment 1. Timing of hatching, morphology, growth/development trade‐offs, and carry‐over effects on tadpole fitness

**FIGURE 1:**
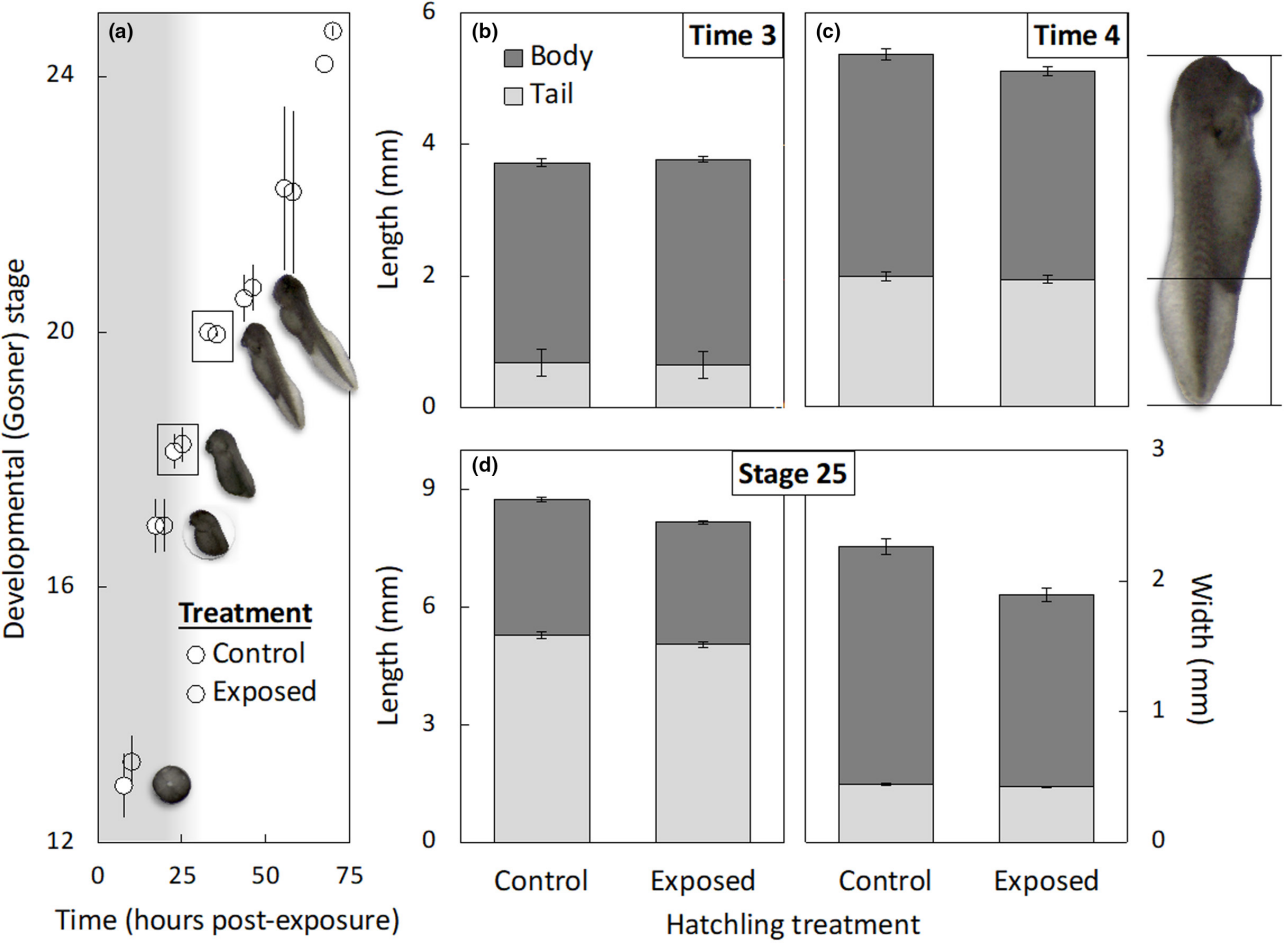
Effect of embryonic exposure to cannibal tadpole cues on development and growth of cane toad eggs and hatchlings through to tadpole stage 25. The developmental stage of eggs and hatchlings from the cannibal‐exposed treatment did not differ from that of their cannibal‐naïve siblings until late in hatchling development (i.e., after 69 h exposure, ~stage 24–25; panel a). However, effects of cannibal exposure on hatchling size were evident before this time. At time 3 (after 24 h exposure, ~stage 18), hatchling size did not differ between control and exposed treatments (panel b). But by time 4 (after 34 h exposure, stage 20), hatchlings from the cannibal‐exposed treatment were smaller than their cannibal‐naïve siblings (panel c). At the conclusion of the hatchling stage (i.e., tadpole stage 25), cannibal‐exposed tadpoles were smaller than their cannibal naïve siblings in multiple size and morphological features (panel d; see also Figure 2c for measurement details). In panel (a), the development data that were previously published in DeVore, Crossland, and Shine (2021: Fig. S3) are adapted here for reference; the inset boxes indicate the times at which hatchling morphology measurements were taken in the present study (24 h = ~stage 18 hatchlings, 34 h = stage 20 hatchlings), and the photo insets depict representative control eggs and hatchlings (from Clutch 1, Oombulgurri) at each time period. The grey background indicates the duration of egg stages, whereas the hatchling stages are depicted on a white background. Note that at the start of the experiment, embryos were placed within treatment tanks ~6 h post‐laying, such that the entire duration of pre‐feeding development was ~3 to 3.6 days. All data plotted are mean values ± SE.

In a previous study, we demonstrated that late‐stage hatchlings of invasive cane toads in Australia accelerate their development rates (i.e., Gosner stage) in response to waterborne cues of non‐feeding cannibal tadpoles (DeVore, Crossland, & Shine, 2021). Specifically, hatchlings exposed to conspecific tadpole cues are more likely to have developed to stage 25 (the free‐swimming tadpole stage) after 69 h than their unexposed siblings (*p* = .02, DeVore, Crossland, & Shine, 2021, Figure 1a; the data plotted in Figure 1a are re‐drawn from Fig. S3 of DeVore, Crossland, & Shine, 2021, and are included here for reference). This cannibal‐induced accelerated rate of development is not evident during the egg or early hatchling stages (*p* > .10, DeVore, Crossland, & Shine, 2021; i.e., there is no evidence of accelerated development at time of hatching). Note that, in addition, DeVore, Crossland, and Shine (2021) demonstrated that cannibal‐induced carry‐over effects reduced the mean growth, development, and survival of these tadpoles 10 days after they had transitioned from the hatchling stage. However, these previous analyses only examined overall treatment effects (control vs. embryonic exposure to cannibal tadpole cues) and did not include morphological measurements or examine the role of size at the end of the hatchling stage as an individual‐level predictor of these negative carry‐over effects. The results for these morphological responses to cannibal cues and their effects on subsequent performance are presented here for the first time.

During this study, we collected data on the effects of cannibal tadpole cues on the (1) time and success of hatching, (2) size and morphology of hatchlings at two time periods, (3) size, morphology, and mouthpart integrity of stage 25 tadpoles, and (4) carry‐over effects of early exposure and size variation on the fitness‐related traits of individually raised tadpoles (i.e., tadpole growth, development, mouthpart integrity, survival). Here, we present the previously unpublished analyses of these data. Importantly, the data we present for Experiment 1 in the current paper are for the same individual hatchlings and tadpoles measured for developmental acceleration and mean carry‐over effects in DeVore, Crossland, and Shine (2021). Thus, combining our new data and analyses on morphology with the developmental acceleration results of DeVore, Crossland, and Shine (2021) allowed us to explicitly assess development/growth trade‐offs during the hatchling stage in response to exposure to cannibal tadpole cues and determine whether the mean effects observed in these clutches are related to individual‐level variation in size at the end of the hatchling stage.

Full experimental details are given in DeVore, Crossland, and Shine (2021). Briefly, for each of two egg clutches (source population: Oombulgurri), we filled three 1 L containers with 750 mL water to act as treatment water sources. One container remained tadpole‐free and served as the control. In each of the remaining two containers, we placed two tadpoles (source populations: Ellenbrae and Palm Creek [one clutch each]; tadpoles from different populations placed in separate containers). All containers were allowed to sit for 2 h to generate cues, before aliquots were drawn for exposure to hatchlings as described below. All containers were located in a shaded open‐air facility (mean temperature ~25°C).

We allocated nine petri dishes to each egg clutch (three control replicates and three replicates per cannibal tadpole clutch). We pipetted 50 mL water from the appropriate treatment water source (control, two cannibal tadpole clutches) into each petri dish. We then randomly allocated five early‐stage eggs (stage 4–6) to each petri dish and allowed them to develop, with 100% water change from source treatment containers every 10–12 h. Chemical cues from the tadpole clutches were therefore present in the cannibal‐exposed treatments, even though cannibal tadpoles were not physically present.

We visually monitored eggs and hatchlings using a stereo microscope with camera attached. To assess timing of hatching, we photographed developing eggs leading up to, and throughout, the hatching process (clutch 1: 8 h = egg stage 9–13, 19 h = egg stage 15–16, 24 h = hatchling stage 17–18; clutch 2: 10 h = egg stage 12–14, 18 h = hatchling stage 17–18, 24 h = hatchling stage 18). Hatching was defined as complete emergence from the egg jelly string and interior egg capsule. To assess changes in body size and morphology, we continued to monitor these hatchlings at 10–12 h intervals until they reached the free‐swimming tadpole stage. We photographed all hatchlings at the 3rd and 4th monitoring periods (i.e., ~stage 18 and 20), as well as at the point at which each hatchling transformed into a free‐swimming, feeding tadpole (stage 25; note that the timing of this transition varied between individuals—see below for further details). All photographs included a grid for size reference. We also scored tooth row keratinisation for each stage 25 tadpole: mouthparts were evaluated by a single observer by using a wide pipette to gently turn live tadpoles on their backs and view them under a dissector microscope, and given a value ranging from 0 (no keratinisation of mouth papillae or tooth rows; i.e., 0% keratinisation) to 4 (fully keratinised papillae and tooth rows; i.e., 100% keratinisation). The cannibal‐exposed individuals in this experiment significantly increased their developmental rate to stage 25 (DeVore, Crossland, & Shine, 2021; Figure 1a); thus, any cannibal treatment effects on hatchling size/morphology, and subsequent tadpole growth, development, and survival should be interpreted in this context.

Once photographed, each stage 25 tadpole was placed in a labelled 200 mL container holding 150 mL water and transferred to an indoor laboratory (mean temperature ~27°C). The time lag between the first and last hatchling from a given clutch to reach stage 25 and be allocated to its container was 12 h, with all individuals reaching stage 25 on the same calendar day regardless of treatment. These tadpoles were then raised individually (in the absence of cannibal tadpole cues) for 10 days on a diet of powdered algae wafers (Hikari, Kyorin, Japan) fed ad libitum, with water changes every 3 days. We measured survival daily. At day 10, we also measured tadpole mass (0.001 g), development stage, and tooth row keratinisation. Because individual tadpoles were not exposed to conspecific tadpole cues during this 10‐day period (starting at stage 25), any treatment effects on tadpole responses at day 10 represent carry‐over effects of early exposure to conspecific cannibal tadpole cues.

**FIGURE 2:**
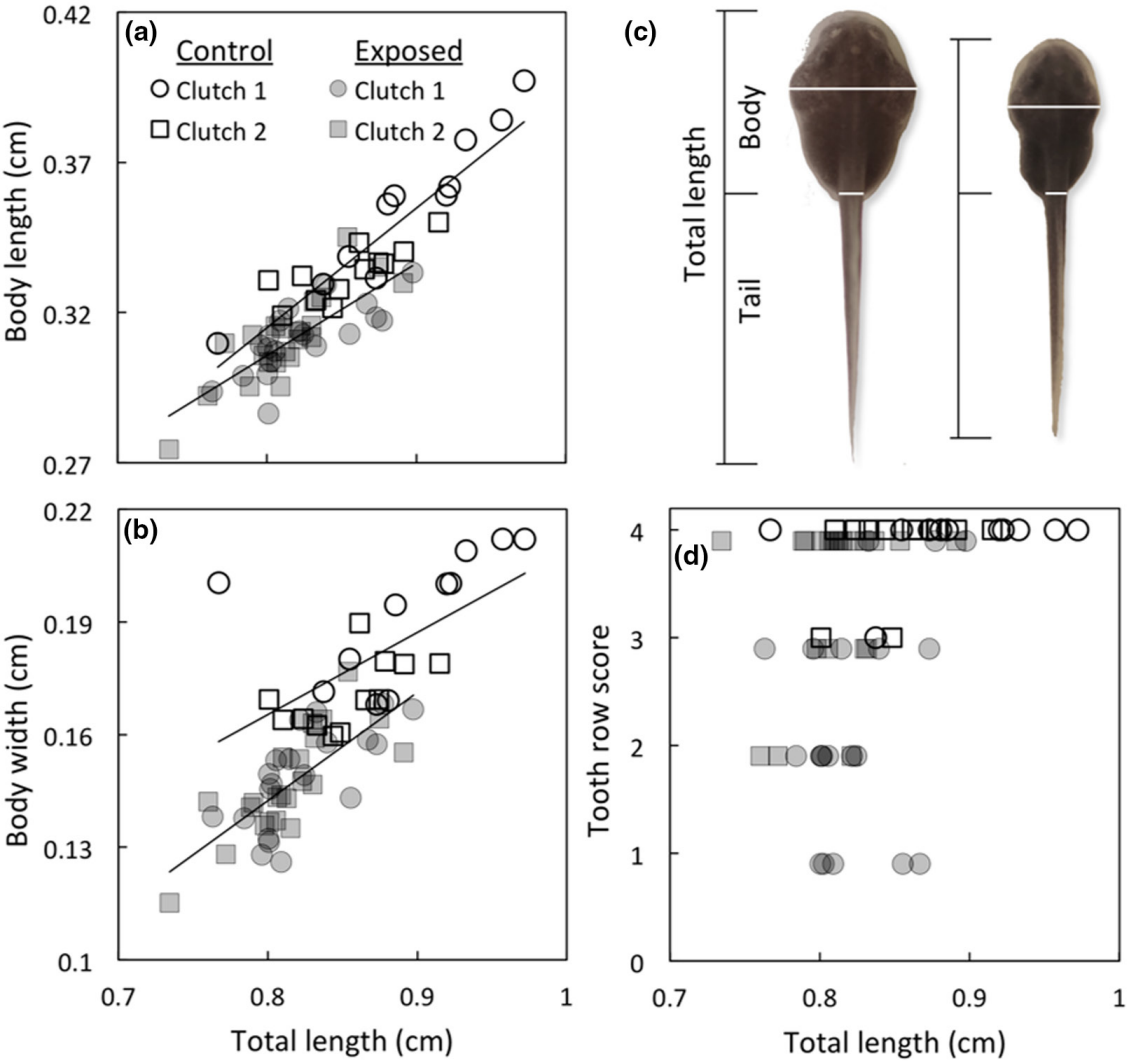
Effect of embryonic exposure to cannibal tadpole cues on morphology of stage 25 cane toad tadpoles. Exposure to cannibal cues during pre‐feeding development affected morphology at the beginning of the tadpole stage such that, for their size, cannibal‐exposed stage 25 tadpoles had shorter (panel a), narrower (panel b) bodies than siblings from control conditions (consequently, these tadpoles also had relatively longer tails). Panel (c) depicts the positioning of these length (black) and width (white) measurements on control (left) and cannibal‐exposed (right) stage 25 tadpoles from Clutch 1 (Oombulgurri). Larger stage 25 tadpoles also had more fully keratinised tooth rows, regardless of treatment (panel d; tooth rows scores ranged from 0 to 4 [i.e., 0%–100% keratinisation; these scores were converted to logit transformed proportions prior to analysis]). The raw values for each tadpole are plotted for panels (a), (b) and (d).

We used ImageJ 1.52q (http://imagej.nih.gov/ij) to measure body length (tip of head to junction of body/tail musculature), tail length (end of body length measurement to tip of tail), and total length (tip of head to tip of tail) for both hatchlings and stage 25 tadpoles (Figures 1 and 2). We also measured body width and tail width for stage 25 tadpoles (Figure 2). For consistency, only individuals positioned in a single plane of body orientation were measured (i.e., individuals with bent body posture in photographs were excluded). This resulted in photographs for 55 stage 18 hatchlings, 60 stage 20 hatchlings, and 65 stage 25 tadpoles being used for analysis. The junction point of the body and tail could not be clearly identified in photographs of six stage 18 hatchlings and two stage 20 hatchlings; for these individuals, we only measured total length.

### 2.3 Experiment 2. Do embryos exhibit plasticity in other developmental traits in response to cannibal cues?

We placed 12 plastic tubs (32 cm × 25 cm × 20 cm) in a shaded open‐air facility and filled each with 8 L water. We then placed a section of egg string containing 15 eggs (stage 10–11) from a single egg clutch (source population: Middle Point) in the centre of each tub. In the corner of each tub we placed a 75 mL plastic container sealed with 1 mm^2^ fibreglass mesh. Each 75 mL container housed either no cannibal tadpoles (control) or three cannibal tadpoles (cannibal cue treatment). Tubs were placed in pairs of one control and one treatment tank, randomly assigned, per spatial block. We used tadpoles from six different clutches to generate cannibal cues, and all caged tadpoles within a given exposure tub were from the same clutch (one tadpole clutch each from Townsville, Ellenbrae, Palm Creek and Kununurra; two clutches from Innisfail).

We photographed developing embryos in each tub from directly above at 0, 24, 36, 45, 54, and 57 h after the start of the experiment. At 0 and 24 h, embryos were still inside the egg strings where they were originally positioned. At 36 h, hatchlings (now ~stage 20) had emerged from egg strings and were in the process of transitioning from lying on their side to an upright body posture, and had also commenced to disperse from the egg strings. This process continued through the 45‐h interval (~stage 21–22). At 54 h, 98% of hatchlings (now ~stage 23) had achieved an upright body posture, and dispersal had increased but hatchlings had not yet reached the sides of the tubs. Dispersal during 36–54 h was short‐distance movements along the bottom of the tub via occasional body twitches. Between 54 and 57 h, many hatchlings reached the sides of the tubs and positioned themselves vertically against the walls at varying heights. This precluded accurate measurement of total distance travelled after 54 h because we were unsure of distance travelled along the sides of the tubs. Therefore, we selected photographs at the 36, 45, and 54 h intervals for analysis of patterns of dispersal distance and body posture.

To assess dispersal patterns, we first highlighted the original position of the egg string in the photographs at 0 h so that as the egg jelly disintegrated over time, we maintained the original reference location. At each subsequent time interval, we measured the distance of each hatchling to the nearest section of the original egg string using ImageJ 1.52q (http://imagej.nih.gov/ij). Because it was not possible to identify which specific hatchling developed from which specific egg, the distance measured for each hatchling is its minimal dispersal distance. To assess patterns in transition of body posture, we scored each hatchling at each time interval as either lying on its side or upright.

We note that, in a separate experiment, we tested the same egg clutch with cues from five of the six same tadpole clutches for sensitivity to cannibal cues and found significant carry‐over effects in the tadpole stage of reduced growth, development, and survival (M. R. Crossland, R. Shine, J. L. DeVore unpublished data). These carry‐over effects can be interpreted as indicative of cannibal‐induced developmental acceleration during the hatchling stage (as demonstrated in DeVore, Crossland, & Shine, 2021). Thus, the egg clutch tested for plasticity in dispersal and body posture responses exhibited cannibal‐induced developmental plasticity in developmental rate.

### 2.4 Statistical analyses

We conducted all analyses in R (R Core Team, 2021). Binomial response variables were analysed using logistic regression (Warton & Hui, 2011) and quasi‐binomial models to account for overdispersion (mixed effects models: package MASS:glmmPQL, Venables & Ripley, 2002). Continuous response variables were analysed using linear mixed‐effects models (package nlme:lme, Pinheiro et al., 2021). Linear mixed‐effects models are particularly useful for analysing ecological (and evolutionary) datasets because they are robust to violations in distribution assumptions that are a common feature of such datasets (Schielzeth et al., 2020).

We conducted all statistical tests at alpha = .05. Further details for specific statistical analyses are given below.

#### 2.4.1 Experiment 1. Effect of cannibal cues on hatching success and timing of hatching

We analysed overall hatching success (total number of eggs to hatch vs. not hatch) using treatment (control vs. cannibal cue) as a fixed effect and egg clutch as a random effect. All eggs that hatched were viable and developed into stage 25 tadpoles.

Some eggs failed to complete development to hatching, resulting in there being less than five hatchlings per container. For eggs that did successfully develop to hatching, we analysed the effect of treatment on timing of hatching as follows. Because each experimental container was checked for the number of hatched eggs at three time intervals (median observation time = 9, 18.5, and 24 h), the data obtained were number of viable eggs that had hatched versus not yet hatched in each experimental container at each time interval. Therefore, we analysed time of hatching for viable eggs using treatment (control vs. cannibal cue) and time (*N* = 3 time intervals) as fixed effects. Exposure dish was nested within egg clutch as a random effect in this model.

#### 2.4.2 Experiment 1. Effect of cannibal cues on size and morphology of hatchlings

For hatchling measurements taken after 24 and 34 h of cannibal‐exposure (~stage 18 and 20, respectively), we analysed total length, body length, and tail length as a response to the fixed effect of treatment (control vs. cannibal cue) at each of these time periods. To determine whether exposure to cannibal cues affected hatchling morphology, we also compared body length between treatments by including total length as a covariate (to correct for individual variation in total length). That is, for body length models that include total length as a covariate, we refer to the model as “relative body length”, meaning body length relative to total length. We initially included the interaction of treatment × total length in these models, however this predictor was not significant (*p* > .14 in both cases) and so was excluded from the final models. In all models, we accounted for random effects by nesting exposure dish within egg clutch.

#### 2.4.3 Experiment 1. Effect of cannibal cues on size and morphology at the conclusion of the hatchling stage

We took developmental stage 25 as the stage at which hatchlings transition into tadpoles because this is the stage they become fully mobile and commence feeding on external food sources (Gosner, 1960; M. R. Crossland, R. Shine, J. L. DeVore personal observation). Once the hatchlings reached this stage, we analysed total length, body length, tail length, body width, tail width, and tooth row keratinisation as a response to the fixed effect of treatment (control vs. cannibal cue). Tooth keratinisation scores were converted to logit proportions prior to analysis. To determine whether early exposure to cannibal cues affected morphology at this stage, we then used separate models to analyse the fixed effect of treatment on the relative response of these variables by including total length as a covariate in each model (i.e., responses relative to total length). For these analyses, we initially included the interaction between treatment × total length. However, none of these interactions were significant (*p* > .14 in all cases) so this interaction was removed from the final models. We accounted for random effects in all analyses by nesting exposure dish within egg clutch.

#### 2.4.4 Experiment 1. Effect of treatment and initial size at stage 25 on subsequent tadpole fitness traits

We used separate models to determine whether, following 10 days in individual containers, tadpole growth (mass), development, tooth row keratinisation or survival were related to the fixed effect of historic egg/hatchling treatment (control vs. cannibal cue). Tooth keratinisation scores were converted to logit proportions prior to analysis. We also tested for treatment effects on the relative response of these variables by including total length at stage 25 as a covariate in each model. The fixed effect of tooth row keratinisation at stage 25 was also included in the relative tooth keratinisation model. We tested for interactions between treatment × total length at stage 25, and removed the interaction where it was not significant. Ultimately, the interaction was removed from the growth, development, and tooth row keratinisation models (*p* > .15 in all cases) but retained for the survival model (*p* = .02; Table 5). Hatchling exposure dish was nested within egg clutch as a random effect in each model to account for non‐independence of tadpoles that, as hatchlings, were raised together within a single container prior to reaching stage 25. We note that the analyses for relative responses at day 10 are new analyses of data presented in DeVore, Crossland, and Shine (2021) that now include the effect of initial size (total length) at stage 25 as an explanatory variable. The role of total length at stage 25 as a predictor of future tadpole fitness has not been assessed previously.

#### 2.4.5 Experiment 2. Effect of cannibal cues on hatchling dispersal and transition to upright body posture

We analysed hatchling dispersal distance and body posture transition (lying on side vs. upright) using treatment (control vs. cannibal cues) and time (36, 45, and 54 h) as fixed effects, and experimental tub nested within block as a random effect. Dispersal data were log‐transformed prior to analysis to improve normality of residuals (package moments, Komsta & Novomestky, 2015: raw data residuals skewness = 4.67 vs. log‐transformed data residuals skewness = −0.27). At 36 and 45 h, some hatchlings had not yet dispersed from their original location within the egg string, resulting in a distance measurement for these individuals of 0. We therefore added a value of 1 to all distance measurements to allow for analysis of log‐transformed data.

## 3 RESULTS

### 3.1 Experiment 1. Effect of cannibal cues on hatching success and timing of hatching

There was no significant effect of exposure to cannibal cues on hatching success (*t* = 0.62, df = 15, *p* = .55; % mean hatching success [lower and upper SE]: control = 80.5 [70.3, 87.9] vs. cannibal cue = 85.5 [78.3, 90.6]). Timing of hatching also did not vary significantly in response to cannibal cues (*t* = 0.39, df = 15, *p* = .71) but, as expected, time itself was a significant covariate (*t* = 9026.40, df = 35, *p* < .0001).

For egg clutch 1, 34 of the 45 embryos hatched, with all hatching occurring after 19 h but before 24 h (i.e., between stage 15–16 and stage 17–18; Table 1). For egg clutch 2, 41 of the 45 embryos hatched; hatching was first recorded at 18 h (stage 17–18), with all hatching completed by 24 h (stage 18; Table 1).

**TABLE 1:** Hatching of cane toad eggs in control versus conspecific tadpole cue treatments.

Egg clutch	Time (h)	Gosner (1960) stage	Control—number viable eggs not yet hatched	Control—number viable eggs hatched	Tadpole cue—number viable eggs not yet hatched	Tadpole cue—number viable eggs hatched
1	8	9–13	11	0	23	0
1	19	15–16	11	0	23	0
1	24	17–18	0	11	0	23
2	10	12–14	13	0	28	0
2	18	17–18	3	10	2	26
2	24	18	0	13	0	28

### 3.2 Experiment 1. Effect of cannibal cues on size and morphology of hatchlings

There was no significant effect of cannibal cues on size of hatchlings after 24 h exposure (~stage 18; mean stage = 17.9, range = 17–18) in terms of body length or tail length; consequently, total length (i.e., body + tail length) was also not affected by treatment (Table 2; Figure 1b). There was also no significant treatment effect on relative body length (Table 2; Figure 1b). Body length was significantly correlated with total length in the relative body length model (Table 2).

**TABLE 2:** Effect of exposure to cannibal tadpole cues on size and morphology of ~stage 18 hatchlings.

Stage 18 hatchlings	*t*	df	*p*	Effect size ± SE
Single main effects				
Total length	0.37	14	.72	0.04 ± 0.10
Body length	1.20	14	.25	0.09 ± 0.08
Tail length	−1.03	14	.32	−0.04 ± 0.04
Relative body length				
Treatment (exposed)	1.85	14	.09	0.06 ± 0.03
Total length	11.21	31	<.0001	0.70 ± 0.06

*Note*: All length data are in mm; relative body length is body length relative to total length at stage 18; refer to Figure 1b.

After 34 h of exposure, cannibal‐exposed hatchlings (stage 20) were smaller in body length (and as a result, total length) than controls but did not differ in tail length (Table 3; Figure 1c). However, after correcting for total length, the relative body length of exposed stage 20 hatchlings did not differ significantly from that of their control siblings, such that exposed hatchings were smaller than control hatchings but morphologically similar (Table 3).

**TABLE 3:** Effect of exposure to cannibal tadpole cues on size and morphology of stage 20 hatchlings.

Stage 20 hatchlings	*t*	df	*p*	Effect size ± SE
Single main effects				
Total length	−2.34	15	.03	−0.26 ± 0.11
Body length	−2.79	15	.01	−0.21 ± 0.08
Tail length	−0.74	15	.47	−0.04 ± 0.06
Relative body length				
Treatment (exposed)	−1.54	15	.15	−0.06 ± 0.04
Total length	9.40	39	<.0001	0.59 ± 0.06

*Note*: All length data are in mm; relative body length is body length relative to total length at stage 20; refer to Figure 1c.

### 3.3 Experiment 1. Effect of cannibal cues on size and morphology at the conclusion of the hatchling stage

Stage 25 tadpoles that had been exposed to cannibal cues during embryonic development were smaller than their unexposed siblings in terms of body length and tail length (and therefore, total length), as well as body width and tail width (Table 4; Figure 1d). There were also significant morphological differences in response to treatment: for their size (total length), cannibal‐exposed stage 25 tadpoles had shorter, thinner bodies and longer tails than did control tadpoles (Table 4; Figure 2a–c). The size of each of these morphological features was also correlated with the total length of the tadpole (Table 4). There was no significant treatment effect on relative tail width at stage 25, such that tadpoles of a given size had similar tail widths, regardless of treatment (Table 4).

**TABLE 4:** Effect of exposure to cannibal tadpole cues on size and morphology at the completion of the hatchling stage (i.e., at stage 25 tadpole).

Stage 25 tadpoles	*t*	df	*p*	Effect size ± SE
Single main effects				
Total length	−4.05	13	.001	−0.55 ± 0.14
Body length	−5.73	13	<.0001	−0.33 ± 0.06
Tail length	−2.32	13	.04	−0.22 ± 0.10
Body width	−7.19	13	<.0001	−0.34 ± 0.05
Tail width	−2.84	13	.01	−0.02 ± 0.01
Tooth keratinisation	−2.65	15	.02	−1.75 ± 0.66
Relative body length				
Treatment (exposed)	−3.81	13	.002	−0.13 ± 0.04
Total length	13.96	48	<.0001	0.36 ± 0.03
Relative body width				
Treatment (exposed)	−5.92	13	.0001	−0.20 ± 0.04
Total length	7.07	48	<.0001	0.24 ± 0.03
Relative tail length				
Treatment (exposed)	3.81	13	.002	0.13 ± 0.04
Total length	25.11	48	<.0001	0.64 ± 0.03
Relative tail width				
Treatment (exposed)	−0.91	13	.38	−0.01 ± 0.01
Total length	3.06	47	.004	0.03 ± 0.01
Relative tooth keratinisation				
Treatment (exposed)	−1.12	13	.28	−0.84 ± 0.75
Total length	3.63	47	.001	1.81 ± 0.50

*Note*: All length and width data are in mm; tooth keratinisation is logit proportion; relative responses are relative to total length at stage 25; refer to Figures 1d and 2a–d.

Cannibal‐exposed stage 25 tadpoles also had reduced tooth row keratinisation compared to controls (Table 4). However, when initial size (total length) at stage 25 is included as a covariate in the model, initial size is a significant predictor of tooth row keratinisation but there is no longer a significant effect of cannibal cue treatment (Table 4). That is, differences in total length apparently underlie the overall effect of treatment on tooth row keratinisation at stage 25: smaller tadpoles had less tooth row keratinisation, and cannibal‐exposed tadpoles were, on average, smaller than control tadpoles (Figure 2d).

### 3.4 Experiment 1. Effect of treatment and initial size at stage 25 on subsequent tadpole fitness traits

**FIGURE 3:**
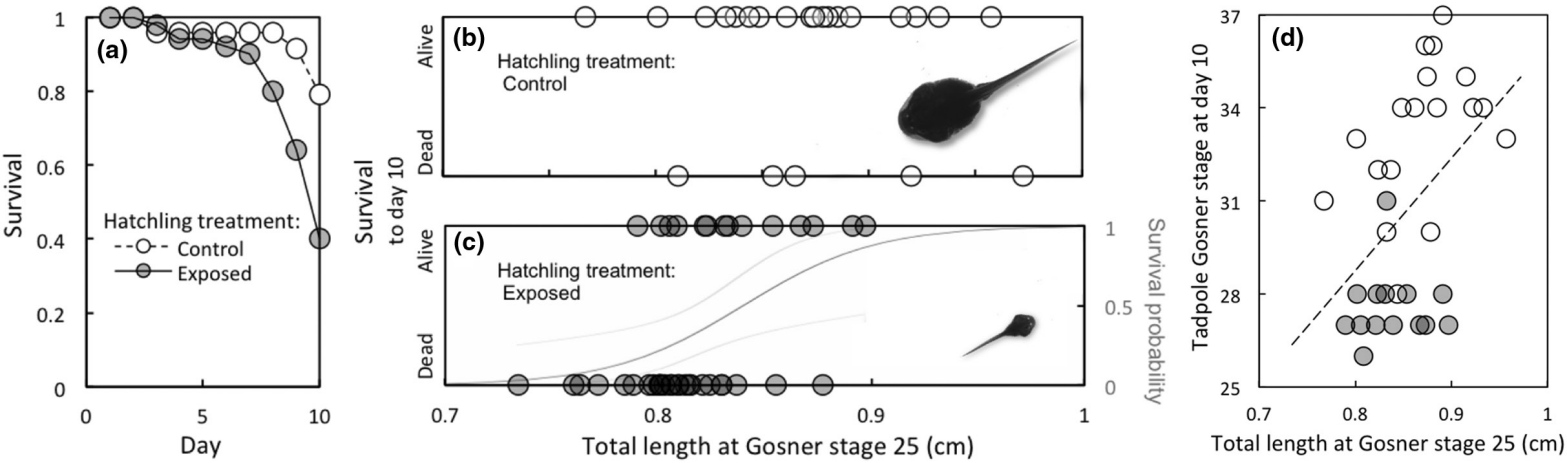
Effect of embryonic exposure to cannibal tadpole cues on cane toad survival and development 10 days after transitioning to the tadpole stage. On average, exposure to cannibal cues during pre‐feeding development reduced tadpole survival (panel a). However, the odds that an individual tadpole would survive were affected by a significant interaction between treatment and initial size (total length) at stage 25. Within control conditions, initial size at stage 25 did not affect subsequent survival (panel b). However, in the cannibal‐exposed treatment, larger stage 25 tadpoles were more likely to survive (panel c; the grey line depicts the relationship between initial tadpole size and the probability of surviving; logistic regression ± 95% CI). The photo insets depict median stage tadpole siblings (Clutch 1, Oombulgurri) from control (b) and exposed (c) treatments at day 10. Tadpoles from control conditions also developed more quickly than those that had been exposed to cannibal cues (panel d; *p* < .0001). In addition, development was marginally associated with initial size, such that individuals that were larger at the beginning of the tadpole stage tended to develop more quickly (panel d; *p* = .06, the dashed line is a simple linear regression illustrating this trend). Note that 73% of the tadpoles <8.1 mm (*N* = 22) died before day 10, and development data are not available for deceased individuals. Thus, significant mortality effects likely constrain interpretation of initial size effects on growth and development at day 10.

Overall, exposure to cannibal cues during embryonic stages significantly reduced growth (mass), development, and tooth row keratinisation in surviving tadpoles at day 10 (Table 5). Survival was also significantly reduced (Table 5; Figure 3a), such that the odds that a control tadpole would survive the first 10 days of the tadpole stage were 5.70 times those of a tadpole exposed to cannibal cues during embryonic development (odds ratio SE = 3.17–10.26).

**TABLE 5:** Effect of embryonic exposure to cannibal tadpole cues on growth (mass), development (stage), tooth row keratinisation and survival of tadpoles 10 days after transforming into stage 25 tadpoles.

Tadpoles at day 10	*t*	df	*p*	Effect size ± SE
Single main effects				
Mass	−7.24	13	<.0001	−38.97 ± 5.38
Development stage	−9.88	13	<.0001	−5.97 ± 0.60
Tooth keratinisation	−6.39	15	<.0001	−7.99 ± 1.25
Survival	−2.96	15	.01	0.18 (0.10, 0.32)^a^
Relative mass				
Treatment (exposed)	−5.76	11	.0001	−32.39 ± 5.62
Total length stage 25	1.96	17	.07	12.64 ± 6.44
Relative development stage				
Treatment (exposed)	−7.39	11	<.0001	−5.17 ± 0.70
Total length stage 25	2.00	17	.06	1.61 ± 0.80
Relative tooth keratinisation				
Treatment (exposed)	−4.54	13	.001	−6.59 ± 1.45
Total length stage 25	2.45	25	.02	3.85 ± 1.58
Tooth keratinisation stage 25	−0.22	25	.83	−0.08 ± 0.36
Relative survival				
Treatment (exposed)	−2.52	13	.03	
Total length stage 25	−0.65	46	.52	
Treatment × total length stage 25	2.43	46	.02	

*Note*: Mass is mg; development is Gosner (1960) stage; tooth keratinisation is logit proportion; survival is proportion alive; relative responses are relative to initial size (total length, mm) at stage 25; refer to Figure 3a–d.

^a^ Effect size for survival is the odds ratio (lower and upper SE).

There was a significant interaction between treatment and initial size (total length) at stage 25 for survival at day 10 (Table 5). Within control treatment containers, initial size was not a significant predictor of probability of survival at day 10 (*t* = −0.62, df = 16, *p* = .54; Figure 3b). However, within cannibal‐exposed treatment containers, initial size was positively associated with probability of survival at day 10 (*t* = 2.61, df = 30, *p* = .01; odds ratio per mm: 37.36, SE = 9.33–149.63): cannibal‐exposed individuals that were smaller at stage 25 were more likely to die than were their larger exposed siblings (Figure 3c).

When initial tadpole size at stage 25 is included in the model, day 10 tadpoles that had been exposed to cannibal cues during embryonic development still had significantly reduced growth (mass) and development (stage) compared to controls (Table 5). The effect of initial size at stage 25 in these growth and development models was marginally non‐significant (*p* = .06–.07; Table 5), although larger initial size tended to be associated with larger mass and stage at day 10 (Table 5; Figure 3d; see Section 4 for interpretation of marginal non‐significance).

After 10 days of growth, tadpole tooth row keratinisation was still significantly related to initial size at stage 25; however, there was now an additional, negative effect of embryonic exposure to cannibal cues on tooth row keratinisation (Table 5). In contrast, tooth row keratinisation at day 10 was not related to tooth row keratinisation at stage 25 (Table 5).

### 3.5 Experiment 2. Effect of cannibal cues on hatchling dispersal and transition to upright body posture

**FIGURE 4:**
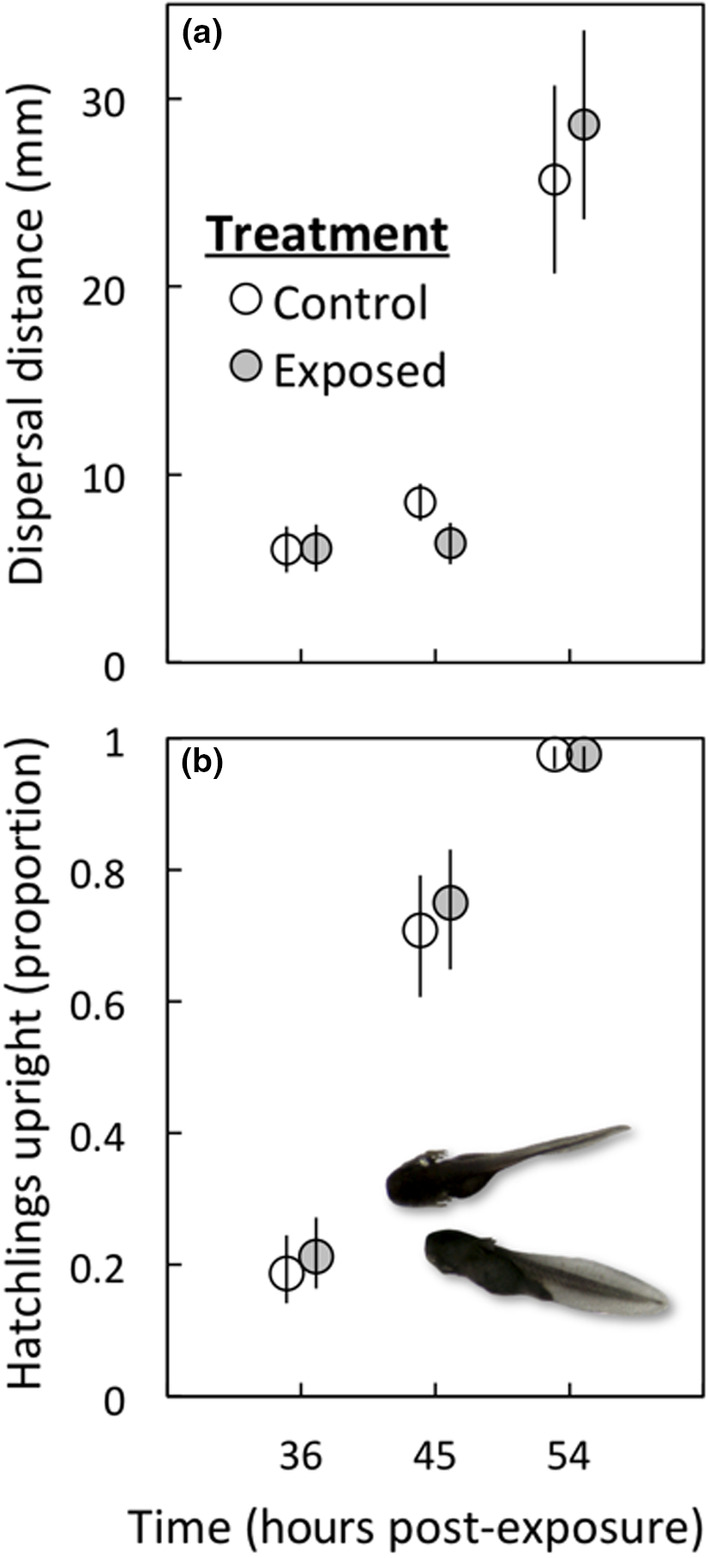
Effect of embryonic exposure to cannibal tadpole cues on dispersal and transition to upright body posture for cane toad hatchlings. Exposure to cannibal cues did not affect (a) hatchling dispersal from the egg string or (b) the proportion of hatchlings upright at time 36 h (~stage 20), 45 h (~stage 21–22), or 54 h (~stage 23). Mean ± SE values are depicted at each time period. The photo inset in panel (b) depicts a hatchling lying prone on its side (lower photo; lateral view) and a hatchling that has transitioned to an upright body posture (upper photo; dorsal view). Photo source is Clutch 1, Oombulgurri.

There was no significant effect of exposure to cannibal cues on either dispersal distance from the egg string or transition to upright body posture (Table 6; Figure 4a,b). As expected due to natural development processes, there was a significant effect of time on both of these hatchling responses (Table 6; Figure 4a,b).

**TABLE 6:** Effect of exposure to cannibal tadpole cues on hatchling dispersal distance from egg string and hatchling transition to upright body posture.

Hatchling response	*t*	df	*p*	Effect size ± SE
Dispersal distance				
Treatment (exposed)	0.04	5	.97	0.01 ± 0.11
Time	12.89	455	<.0001	
Upright posture				
Treatment (exposed)	0.54	5	.61	1.19 (0.86, 1.64)^a^
Time	9.76	22	<.0001	

*Note*: Dispersal distance is log‐transformed distance (mm, see text); upright posture is proportion hatchlings upright; refer to Figure 4a,b.

^a^ Effect size for proportion of hatchlings in upright body posture is the odds ratio (lower and upper SE).

## 4 DISCUSSION

Our results clarify the mechanisms and costs of facultative developmental plasticity for cane toad hatchlings responding to cannibalism risk. We found no evidence that embryos exposed to non‐feeding cannibal tadpole cues alter the timing of hatching (but see below for discussion). Additionally, hatchlings did not change their rates of dispersal from the egg string or the time at which they transitioned to an upright body posture in response to these cues. However, size and morphology were significantly affected by exposure to cannibal cues. Cannibal‐exposed hatchlings at stage 20 were smaller than their cannibal‐naïve siblings, and at the completion of the hatchling stages (i.e., at the transition to stage 25 tadpole), cannibal‐exposed individuals had relatively smaller body lengths and widths, and relatively longer tails, compared to controls. These size changes indicate that the developmental acceleration exhibited by these hatchlings as an adaptive response to cannibal cues (Figure 1a) likely occurred as a trade‐off with hatchling growth. The effect of cannibal‐induced size reduction of hatchlings carried over to the subsequent tadpole life stage; cannibal‐exposed individuals that were smaller at the conclusion of the hatchling stage were more likely to die as tadpoles, and initial size (total length) at the conclusion of the hatchling stage was positively associated with tadpole growth and development (respectively, *p* = .07 and *p* = .06; see below for further discussion) and tooth row keratinisation (*p* = .02) across treatments. However, size reductions at the conclusion of the hatchling stage only partially explained the subsequent performance reductions observed in tadpoles that had been exposed to cannibal cues, indicating that there are additional hidden physiological costs associated with this plastic response beyond the documented effects of initial size.

Our experimental design assessed direct effects and carry‐over effects in response to waterborne chemical cues from cane toad tadpoles that are highly cannibalistic but that were not fed while exposed to developing embryos. For many anuran species, predator diet (including starvation) is a significant factor determining the magnitude of larval responses to predator chemical cues (Schoeppner & Relyea, 2009). However, for cane toads in Australia, feeding of cannibal tadpoles is not required to induce accelerated developmental of hatchling prey (DeVore, Crossland, & Shine, 2021; Figure 1a), nor is it required to induce significant negative carry‐over effects on subsequent growth, development, and survival (Clarke et al., 2015, 2016; Crossland & Shine, 2012; DeVore, Crossland, & Shine, 2021; DeVore, Crossland, Shine, & Ducatez, 2021; McCann et al., 2020). For cane toads, waterborne cues from the tadpoles inherently convey a signal of significant risk of cannibalism to developing embryos. Nonetheless, the question arises, would embryo responses and carry‐over effects be even stronger if cane toad embryos were exposed to conspecific tadpoles that are consuming conspecific eggs/hatchlings? This is not yet clear because the effect of these combined cues has not been assessed. Both cane toad embryos and tadpoles experience significant reduction in future growth, development, and survival when exposed to conspecific alarm chemicals (macerated conspecific tadpoles: Clarke, 2014; Crossland et al., 2019). Thus, the potential exists for cane toad embryos to respond to alarm cues released by conspecific eggs and hatchlings being consumed. However, we note that because all cane toad tadpoles in Australia tested to date are strongly cannibalistic on hatchlings (Crossland et al., 2022; DeVore, Crossland, & Shine, 2021; DeVore, Crossland, Shine, & Ducatez, 2021), it probably would not benefit these hatchlings to differentiate the diet of older conspecific tadpoles: the mere presence of these conspecifics indicates an immediate risk of cannibalism. Nonetheless, future studies could explore this possibility.

A variety of amphibian species have been reported to alter time of hatching in response to predation risk, although responses are inconsistent among taxa (Van Buskirk, 2016). Species that hatch early in response to predation risk typically innately hatch relatively late in development, after hatching competence has been reached (stage 20–25: Capellán & Nicieza, 2007; Chivers et al., 2001; Delia et al., 2019; Gomez‐Mestre et al., 2008; Laurila et al., 2002; Poo & Bickford, 2014; Warkentin, 1995). These species therefore have the capacity to hatch early (i.e., at an earlier developmental stage, often by mechanically breaking free from the egg capsule) but still be sufficiently developed to cope with post‐hatching life. We found no evidence of early hatching of cane toads in response to cannibal tadpole cues, although the time interval between our observations during the time of hatching (5–8 h) may have masked small but nonetheless significant treatment effects on hatching time. For cane toads, early hatching (in response to any egg mortality risk cue) may not be possible because these embryos inherently hatch relatively early at stage 17–18 (Crossland & Shine, 2011; DeVore, Crossland, & Shine, 2021; present study). At these stages, toad hatchlings lack transparent corneas or fully developed gills, and are essentially immobile other than for spasmodic body convulsions (Gosner, 1960; M. R. Crossland, R. Shine, J. L. DeVore personal observation), such that it is likely that hatching in this species occurs as soon as hatching competence is reached. In any event, early hatching in response to cannibal tadpole cues would presumably be disadvantageous for cane toad embryos given that, while these embryos remain within the egg jelly, they do not leach the chemical cues (maternally invested toxins) that attract cannibal conspecific tadpoles (Crossland et al., 2021, 2022; Crossland & Shine, 2011). Delayed hatching in response to predation risk has also been reported for some amphibian species (Gazzola et al., 2015; Moore et al., 1996; Schalk et al., 2002; Sih & Moore, 1993). Delayed hatching can occur if embryos slow their developmental rate, thereby hatching at the same developmental stage (but at a later time), or if developmental rates remain consistent but hatching occurs at a more advanced developmental stage. Ultimately, this strategy has the potential to reduce the risk of cannibalism for cane toad embryos by delaying the moment embryos are detected by cannibal tadpoles. However, we also found no evidence that exposure to tadpole cues results in delayed hatching time (although, again, our 5–8 h observation interval may have precluded detecting a small but significant delay). This apparent lack of delayed response may be due to several factors. First, hatching of anuran embryos is initiated following the development of frontal glands that chemically degrade the egg jelly layers (Altig & McDiarmid, 2007). In another bufonid, *Bufo japonicus*, these hatching glands appear at stage 19 (Yamasaki et al., 1990) but must develop slightly earlier in *R. marina* which hatch at stage 17–18. It may be that the timing of the development of these frontal glands in cane toads cannot be de‐coupled from other developmental processes, such that selective forces cannot act independently upon them. Alternately, delayed hatching may be costly in other ways and so not be favoured by selective pressures. For example, some anuran embryos that delay hatching are unable to thermoregulate, and experience reduced larval growth as a result (Anderson & Petranka, 2003). Finally, although the chemical cues released during hatching actively attract cannibalistic cane toad tadpoles, these tadpoles also consume conspecific eggs within the jelly string when they encounter them. As both egg and hatchling life stages are vulnerable to cannibalism, shifts in the timing of hatching may not substantially reduce risk, and therefore may not be strongly favoured in response to selective pressure from cannibalism.

The accelerated development of cane toad hatchlings to escape cannibalism risk (DeVore, Crossland, & Shine, 2021; Figure 1a) is apparently a rare response among the pre‐feeding stages of amphibians. Whereas predator‐induced shifts in hatching time have been observed in a variety of amphibian species, predator‐induced accelerated development during early, pre‐feeding stages has only previously been reported for two other species (frog eggs: *Rana temporaria* vs. planarian predator, Segev et al., 2015; salamander hatchlings: *Hynobius retardus* vs. cannibal conspecifics, Kishida et al., 2015). Predation‐induced plasticity in time of hatching may be an ancient trait for amphibians, having been conserved for ~19–35 million years in the Centrolenidae (Delia et al., 2019) and for ~34–50 million years in the Phyllomedusidae (Gomez‐Mestre et al., 2008). In contrast, developmental acceleration induced by cannibalism risk has evolved as a common plasticity response in Australian cane toads within the past 89 years (DeVore, Crossland, & Shine, 2021; DeVore, Crossland, Shine, & Ducatez, 2021).

In our study, cannibal‐exposed individuals not only had altered morphology, they were also smaller overall, implying that cannibal‐induced accelerated development occurs as a trade‐off with growth. Although growth/development trade‐offs are well known for the tadpole stage of anurans (Bridges, 2002; Kulkarni et al., 2011; Wilbur & Collins, 1973), we believe this system is the first in which such a trade‐off has been demonstrated in the anuran hatchling stage. Many previous studies have reported reduced size for anuran embryos that hatch early in response to predation risk; however, these size reductions are simply a function of age at hatching rather than a trade‐off with growth rate (Capellán & Nicieza, 2007; Chivers et al., 2001; Gomez‐Mestre et al., 2008; Laurila et al., 2002; Warkentin, 1995, 1999). In our study, negative effects of cannibal cues on growth were first evident when hatchlings reached stage 20 (Table 3), considerably before changes in development rate were detected (stage 24–25; Figure 1a) and could not be readily explained by increased energetic investment in early dispersal behaviour in response to cannibalism risk, as hatchling dispersal did not differ between treatments. By the time cannibal‐exposed hatchlings reached stage 25 (i.e., the beginning of the tadpole stage), effects on growth were increasingly evident (Table 4). These growth reductions coincided with detectable acceleration of developmental rate (Figure 1a).

Upon transitioning to the tadpole stage (stage 25), cannibal‐exposed individuals were not only smaller than their unexposed siblings, but were also morphologically distinct, with relatively smaller bodies (body length and width) and relatively longer tails. This morphological response can be induced in tadpoles of a variety of species following exposure to predators (e.g., dragonfly larvae or fish: McCollum & Van Buskirk, 1996; Relyea, 2001) and is thought to facilitate predator escape. While it is possible that these morphological changes are also adaptive in cannibal‐exposed cane toad hatchlings, our data on hatchling dispersal did not reveal any obvious effects of cannibal cue exposure on mobility. However, relatively long tails could facilitate burst swimming if hatchlings are contacted by cannibalistic tadpoles or increase the probability of non‐lethal attacks to the tail rather than the body. Another possibility is that cannibal exposure has influences on movement or dispersal later in hatchling development that we were not able to detect during the earlier hatchling stages. Indeed, behavioural observations of cannibal‐exposed tadpoles taken 24 h after reaching stage 25 have shown that they spend more time surface‐swimming and less time feeding than their naïve siblings, although the adaptive advantage of this behavioural shift is unclear, as toads are no longer vulnerable to cannibalism once they reach this stage (DeVore, Crossland, & Shine, 2021). It is also possible that a small body size relative to tail length is a generalised predator‐induced morphology that begins to be exhibited during relatively immobile, pre‐feeding life stages despite not being advantageous during this life stage, or that this morphology occurs as a result of rapid egg yolk depletion as limited endogenous energy reserves are directed toward accelerating development (Arendt, 1997; Auer et al., 2010; Prasad et al., 2001). Whether or not this morphological plasticity has an adaptive function therefore remains an open question.

The role of size as a predictor of future fitness has been documented in several life history stages of anurans. Egg size can determine larval duration and size at metamorphosis (Berven & Chadra, 1988; Kaplan, 1985), size at hatching can determine vulnerability to predators (Kaplan, 1992; Kishida et al., 2015; Warkentin, 1995), and size at metamorphosis can affect future growth, survival, and size at maturity (Altwegg & Reyer, 2003; Cabrera‐Guzmán et al., 2013; Relyea, 2007). However, few data exist regarding the fitness consequences of body size at the ontogenetic switch point from hatchling to tadpole. Under high food conditions, hatchling size at stage 24–25 has been shown to be a significant predictor of future tadpole growth (negative correlation), larval period (positive correlation) and size at metamorphosis (positive correlation) for *Rana lessonae*, but not for *Rana esculenta* (Semlitsch & Schmiedehausen, 1994). Our data on initial size (total length) of stage 25 cane toad tadpoles demonstrates the critical importance of size at the hatchling/tadpole transition point for predicting future fitness of cannibal‐exposed individuals. After 10 days' growth, cannibal‐exposed tadpoles that were smaller at stage 25 were significantly less likely to survive than their larger cannibal‐exposed siblings. This effect is even more striking when you consider that our 10‐day growth experiment was conducted under ecologically benign conditions of no competitors, no predators, abundant food, and regular water changes to limit build‐up of waste metabolites. There was also a significant, negative carry‐over effect of initial size at stage 25 on tooth row keratinisation at day 10. Carry‐over effects of initial size of exposed individuals on subsequent growth and development at day 10 were also evident, although marginally non‐significant (respectively, *p* = .07 and *p* = .06). However, these latter results need to be interpreted in light of the significant effects of cannibal exposure on survival: the tadpoles that died following exposure to cannibal cues, and thus were excluded from growth/development analyses, were smaller individuals at earlier developmental stage (M. R. Crossland, J. L. DeVore personal observation). Thus, significant survival effects at day 10 may have masked carry‐over effects of initial size on subsequent growth and development.

Although size at the conclusion of the hatchling stage had clear effects on performance during the subsequent tadpole stage, the negative effects of early exposure to cannibal cues exceeded those predicted by growth effects alone. This plastic response is therefore associated with additional physiological costs, beyond immediate growth effects, that manifest during the subsequent life stage (De Block & Stoks, 2008). Accelerated development may be an especially costly inducible response, as developmental acceleration has been shown to cause increased oxidative stress (damage to lipids, proteins and DNA), reduced somatic maintenance, reduced immune system function, and/or reduced telomeres in a variety of organisms (nematodes: Lind et al., 2017; damselfly larvae: De Block & Stoks, 2008; Janssens & Stoks, 2018; anuran tadpoles: Burraco et al., 2017; Gervasi & Foufopoulos, 2008), and has been directly linked to reduced lifespan (Hooper et al., 2017; Janssens & Stoks, 2018; Lind et al., 2017). More broadly, the perception of predation risk is also associated with oxidative stress (Janssens & Stoks, 2013). One or more of these factors may be involved in the survival cost of cannibal‐induced accelerated development in cane toad hatchlings. Additionally, resource depletion may have contributed to these survival reductions, especially since the smaller body size of cannibal‐exposed tadpoles could indicate depleted yolk resources. Given that the smallest tadpoles also exhibited the most pronounced oral deformations (which reduces foraging efficiency), and cannibal exposure is followed by behavioural changes that include reduced feeding (DeVore, Crossland, & Shine, 2021), these tadpoles may never recover from this early disadvantage. Future studies could usefully explore these possibilities.

Finally, we note that our demonstration of a cannibal‐induced trade‐off between growth and development during the hatchling stage and the negative carry‐over effects of this trade‐off is based on two clutches for which we have detailed information. How representative are these responses likely to be for cane toad clutches in general? The first factor to consider is that not all cane toad clutches respond equally to cannibal tadpole cues. DeVore, Crossland, and Shine (2021) assessed cannibal‐induced developmental acceleration (but not growth trade‐off responses) for hatchlings of 13 cane toad clutches and found that, while some clutches exhibited strong developmental acceleration, six clutches did not exhibit a significant response. For clutches that were responsive to cannibal tadpole cues, greater developmental acceleration in the hatchling stage resulted in greater impairment of subsequent growth, development, and survival (i.e., stronger negative carry‐over effects: DeVore, Crossland, & Shine, 2021). Based on the results of our current study, we would predict clutches that exhibited significant developmental acceleration also induced a growth/development trade‐off in the hatchling stage, resulting in reduced size as stage 25 tadpoles, and that this size reduction affected their future fitness traits. However, as the non‐responsive clutches observed in DeVore, Crossland, and Shine (2021) exhibited inherently rapid development that was unaffected by cannibal exposure, these clutches could not exhibit a growth/development trade‐off in response to tadpole cues (although whether these clutches would be expected to exhibit a morphological response, e.g., by developing relatively long tails, is less clear). More broadly, the recent evolution of targeted cannibalistic behaviours in Australian cane toad tadpoles may mean that the variation in cannibal effects among cane toad clutches reflects an evolutionary transition from facultative developmental acceleration to canalised rapid development (DeVore, Crossland, & Shine, 2021; DeVore, Crossland, Shine, & Ducatez, 2021). If so, we may expect the growth/development trade‐off we document here to become less common in the cane toads of Australia over time.

## AUTHOR CONTRIBUTIONS

**Michael R. Crossland:** Conceptualization (equal); data curation (equal); formal analysis (equal); investigation (equal); methodology (equal); writing – original draft (equal). **Richard Shine:** Conceptualization (equal); funding acquisition (lead); methodology (equal); resources (lead); supervision (lead); writing – original draft (equal). **Jayna L. DeVore:** Conceptualization (equal); data curation (equal); formal analysis (equal); investigation (equal); methodology (equal); writing – original draft (equal).

## CONFLICT OF INTEREST STATEMENT

The authors declare no competing interests.

## Data Availability

The data and R script are available on figshare (https://doi.org/10.6084/m9.figshare.23813763).
